# Development of a polygenic score predicting drug resistance and patient outcome in breast cancer

**DOI:** 10.1038/s41698-024-00714-7

**Published:** 2024-10-02

**Authors:** Divya Sahu, Jeffrey Shi, Isaac Andres Segura Rueda, Ajay Chatrath, Anindya Dutta

**Affiliations:** 1 Department of Genetics, University of Alabama at Birmingham, Birmingham, AL 35294 USA; 2 Department of Biochemistry and Molecular Genetics, University of Virginia, Charlottesville, VA 22903 USA

**Keywords:** High-throughput screening, Predictive markers, Prognostic markers

## Abstract

Gene expression profiles of hundreds of cancer cell-lines and the cell-lines’ response to drug treatment were analyzed to identify genes whose expression correlated with drug resistance. In the GDSC dataset of 809 cancer cell lines, expression of 36 genes were associated with drug resistance (increased IC50) to many anti-cancer drugs. This was validated in the CTRP dataset of 860 cell lines. A polygenic score derived from the correlation coefficients of the 36 genes in cancer cell lines, UAB36, predicted resistance of cell lines to Tamoxifen. Although the 36 genes were selected from cell line behaviors, UAB36 successfully predicted survival of breast cancer patients in three different cohorts of patients treated with Tamoxifen. UAB36 outperforms two existing predictive gene signatures and is a predictor of outcome of breast cancer patients independent of the known clinical co-variates that affect outcome. This approach should provide promising polygenic biomarkers for resistance in many cancer types against specific drugs.

## Introduction

Personalized chemotherapy has significantly advanced cancer treatment. Yet, the heterogeneity in patient responses and the prevalence of tumor relapse highlights the critical need for more biomarkers that can predict chemotherapeutic outcomes. Genetic alterations, such as acquired secondary mutations in BRCA1 and BRCA2 genes make breast cancer cells resistant to anti-cancer drugs^1^. Genetic deletion of Snail and Twist transcription factors, responsible for EMT, displayed enhanced sensitivity to antimetabolite drug treatment and increased survival of genetically engineered mouse models^2^. In addition, activation of cancer cell survival signaling pathways^3,4^, copy number variations, loss of DNA damage repair^5^, drug efflux^6^ or transition to cancer stem cells^7^ have been implicated in the development of drug resistance. Nonetheless, these genetic alterations alone are not sufficient to explain the complexity of resistance mechanisms. Drug resistance mechanisms are also dependent on changes in gene expression, and we focused on gene expression levels as a biomarker for drug resistance. Gene expression changes are, of course, caused by both genetic and epigenetic events and thus this approach will later lead to the search for mechanisms of chemoresistance in the epigenetic arena.

Several large-scale cancer cell lines screens such as the Genomics of Drug Sensitivity in Cancer (GDSC)^8–10^ and Cancer Therapeutics Response Portal (CTRP)^11–13^ databases, have systematically treated ~1000 human cancer cell lines with more than 100 anti-cancer drugs and recorded the drug response (IC50) for these cell lines. In parallel, databases like Catalogue of Somatic Mutations in Cancers (COSMIC)^14^ contain the transcriptomics profiles of hundreds of cell lines, many overlapping with the cell-lines studied in GDSC and CTRP. These datasets allow us to investigate whether gene expression levels correlate with drug response in a large panel of cancer cell lines. Using the IC50s for hundreds of cancer cells, there have been some attempts to use the gene expression to predict responsiveness to drugs. Although, a few studies have started leveraging this data to find drug resistance markers^15–17^, none of these studies verified whether the drug efficacy prediction from the cancer cells successfully predicts patient cancer outcome in response to a specific drug, a deficit that we set out to correct.

Here we use correlation analysis of expression levels of protein-coding genes and drug responses to identify a list of 36 genes predictive of resistance in several cancer cell lines across many different lineages to tens of drugs. The broad prediction of resistance across cancer lineages and drugs suggested that multiple drug resistance mechanisms were being covered by this analysis. As proof of principle, we first focused on FAM129B/NIBAN2 as it was the top candidate gene whose high expression correlated with resistance to several FDA approved drugs. Next, we integrated the correlation coefficients and expression levels of FAM129B, and the next 35 genes found in our analysis into a polygenic score and demonstrated that this score can predict tamoxifen resistance in cancer cells. Most important, the score can predict outcome of tamoxifen therapy on (estrogen receptor positive/human epidermal growth factor receptor 2 negative (ER^+^/HER2^−^) breast cancer patients. Furthermore, the score derived from the 36 genes (UAB36) performs better than existing polygenic scores and predicts patient outcome even after taking into account clinical co-variates like age, stage and grade in a multi-Cox regression analysis.

## Methods

### Data acquisition and preprocessing

The drug sensitivity data was obtained from the two large-scale pharmacogenomic databases GDSC and the CTRP, in which many anti-cancer drugs were screened against hundreds of cell lines to determine its sensitivity. For the GDSC dataset, the drug response data of 192 drugs for 809 cancer cell lines were obtained from the GDSC website (ftp://ftp.sanger.ac.uk/pub/project/cancerrxgene/releases/current_release/GDSC2_fitted_dose_response_25Feb20.xlsx). The natural log of the IC50 value was used as a measurement for the drug response. It includes a total of 135,242 IC50 values. The IC50 values are expressed in micromolar concentrations. The 809 cancer cell lines screened correspond to 30 different cancer types. We included 25 cancer types for which at least 9 cancer lines treated with anti-cancer drugs as shown in Supplementary Table 1. The Z-score as a standard score of gene basal expression (untreated) profiles of 16,248 genes for 970 cancer cell lines were obtained from the COSMIC database (https://cancer.sanger.ac.uk/cell_lines/archive-download#:~:text=CosmicCLP_CompleteGeneExpression.tsv.gz) accessed on the 5^th^ January, 2021, where the gene expression had been profiled using Affymetrix Human Genome U219 array platform.

For the CTRP dataset, the drug response of 481 drugs for 860 cancer cell lines and basal gene expression profiles of 18,541 genes across cancer cell lines were obtained from the National Cancer Institute Cancer Target Discovery and Development portal (https://ctd2-data.nci.nih.gov/Public/Broad/CTRPv2.1_2016_pub_NatChemBiol_12_109/). The drug sensitivity was measured as area under the dose-response curve (AUC). It includes a total of 326,149 AUC values. The AUC values in CTRP range from 0 to 29.4. The expression value for each gene was multi-array average (RMA) -normalized and log2-transformed, where the gene expression had been profiled using Affymetrix Human Genome U133 plus 2.0 platform. We scaled the expression of each gene into Z-score across cancer cell lines.

### Correlation of gene expression with drug sensitivity

For each cancer cell line, the expression of the gene in the untreated cells and the corresponding drug response value of that cell line was extracted. In the GDSC database, we investigated the Spearman’s correlation coefficient (SCC) between the standard score (Z-score) of gene expression for each gene to the IC50 value for each drug across 777 cell lines. In the CTRP database, AUCs were provided, measuring the relationship between drug concentration and cell viability. Higher AUCs, like higher IC50, show that a cell line is more resistant to a given drug. We performed SCC between the expression levels of each gene to the AUC values for each drug across 727 cell lines. We hypothesize that if expression of a gene is correlated positively with IC50 or AUC in a large number of cell lines, then the gene could be associated with drug resistance. Conversely, if expression of gene is correlated negatively with IC50 or AUC, then expression of gene could be a marker of sensitivity to the drug.

### Constructing polygenic score for predicting drug resistance in cancer types

We obtained a list of 36 genes which showed statistically significant resistance to 20 or more FDA approved drugs in the pan-cancer analysis. For each cancer type represented by at least 9 cell lines with available data, we performed correlation analysis of each gene Z-score expression and IC50 for the respective drug per cell line to determine the SCC for that gene. This yielded 36 SCCs for the respective drug. The polygenic score of 36 genes, here termed as UAB36, for a given cancer cell (or tumor) was calculated by a linear combination of expression and SCC of the gene as follows:

1 $${{{Polygenic}\,{score}}}_{{drug}}=\mathop{\sum }\limits_{g=1}^{n}S{{CC}}_{g}* {exp}_{{cg}}$$

where SCC_g_ is the spearman correlation coefficient for gene g, exp_cg_ is the expression value of gene g in the cell line c (or tumor c), and n is the number of genes integrated into the polygenic score. Here, n is 36.

We repeated the above approach for 51 FDA approved drugs and therefore, for each cancer type, we obtained 51 FDA-drug specific polygenic score.

### Fisher’s Z-transformation

To stabilize the variance and improve the distributional property of the SCC from skewed distribution to normal distribution, the calculated SCC of IC50 or AUC for each drug versus the Z-score expression of FAM129B or the polygenic score of 36 genes, in each cancer type was transformed into Z-scores using Fisher’s Z- transformation as follows,

2 $$F\left(r\right)=\frac{1}{2}\log \left(\frac{1+r}{1-r}\right)$$

where, *F(r)* is the Fisher transformed score of *r*, the SCC. The Z-score of the Fisher transformed SCC was calculated by the following expression,

3 $$Z=\sqrt{\frac{N-3}{1.06}\,}F(r)$$

where, *Z* is the Z-score of the *r* and *N* is the sample size.

### Gene set enrichment analysis

The RNA-seq expression datasets of TCGA breast cancer (BRCA) and METABRIC breast cancer were used. The expression of each gene was normalized to z-score across samples. Further, the samples were stratified into two groups based on quartile expression of UAB36 as described above. The RNA expression fold changes between these two groups of patients were used to assess what pathways were correlated with UAB36 score in breast cancer using gene set enrichment analysis (GSEA 4_1.0)^18,19^. We used the molecular signature database (MSigDB) gene set collection including MASSARWEH_TAMOXIFEN_RESISTANCE_UP gene set^20^ and CREIGHTON_ENDOCRINE_THERAPY_RESISTANCE_5 gene set^21^, maximum gene set size of 5000, minimum gene set size of 10, total number of 1000 permutations, and weighted enrichment statistics.

### Statistics and reproducibility

The Kaplan–Meier survival analysis was performed for the two groups of patients classified based on quartile gene expression (or UAB36 score) value. Patients in which polygenic score of 36 genes (here; UAB36) expression falls in the first quartile (q1) were classified as low-score group, and in the fourth quartile (q4) as high-score group. The significance of the difference between survival curves was assessed using the log-rank test. The univariate and multivariate Cox proportional hazard regression was performed to determine the prognostic value of UAB36. The significance of the difference between violin plots was assessed using the Wilcoxon rank sum test. The multiple hypothesis correction was performed using the Benjamini–Hochberg method. All statistical tests were two-sided. Measurements were taken from distinct samples. Survival analysis was performed using the *survival* R package^22^. The sample sizes of the GDSC, CTRP, TCGA, METABRIC and GSE9195 were predetermined by their data availability. Cancer cell lines or tumors were not randomized into groups because this was not relevant to this study. We did not calculate sample size using power analysis as it was not relevant to this study. We included 25 cancer types for which at least 9 cancer cell lines treated with anti-cancer drugs in the GDSC dataset. For the TCGA-BRCA (ER^+^/HER2^−^) cohort we included 262 samples. For METABRIC breast cancer (ER+/HER2−) cohort (*n* = 895) and for GSE9195 ER^+^ breast cancer (*n* = 77). This study is exempted from the Institutional Review Board approval because it is a secondary analysis of data downloaded from the public available datasets, as indicated in the following websites: TCGA- Genomics Data Commons (GDC) database (https://www.cancer.gov/ccg/research/structural-genomics/tcga/history/policies/tcga-human-subjects-data-policies.pdf), cBioPortal database (https://groups.google.com/g/cbioportal/c/GhmBJLZMk3A/m/AuWn5BnzAAAJ), and NCBI- Gene Expression Omnibus (GEO) database (https://www.ncbi.nlm.nih.gov/geo/info/disclaimer.html).

## Results

### Discovery pipeline for identification of expression biomarkers associated with drug response

**Fig. 1:**
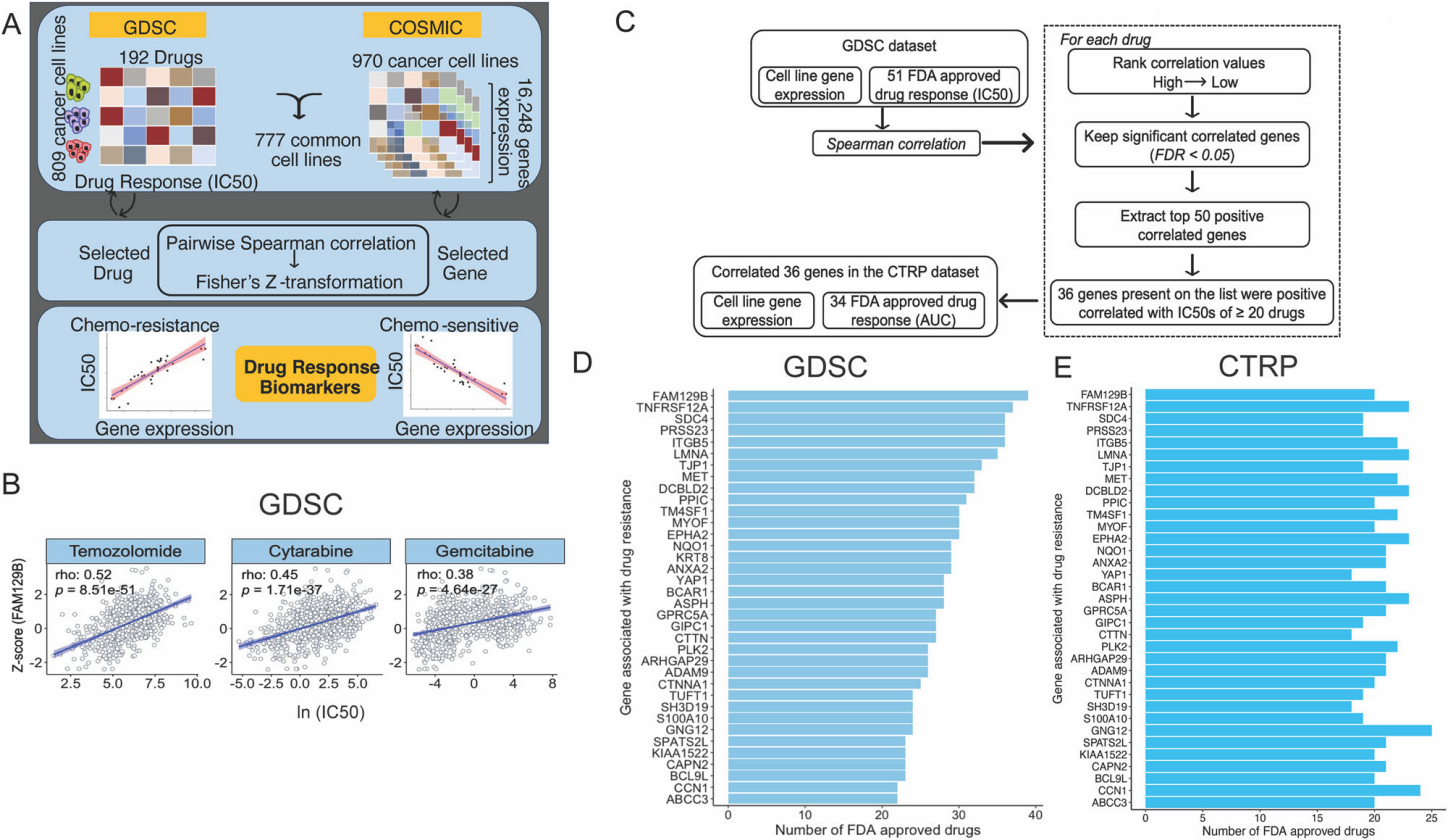
Workflow to identify genes whose expression positively correlated with anti-cancer drug resistance in cancer cell lines. **A** Framework for discovering biomarkers that predict response to anti-cancer drugs. **B** Spearman correlation of IC50s of anti-cancer drugs with z-score normalized expression levels of FAM129B across cancer cell lines (*n* = 777) in the GDSC database. **C** Schematic shows the workflow by which FAM129B, and other drug resistance genes were identified in cancer cell lines for 51 FDA approved drugs from the GDSC and 34 FDA approved drugs from the CTRP database. **D** 36 genes in GDSC that were among the top 50 genes for ≥20 drugs, when ranked by spearman correlation coefficient (SCC) of gene expression with IC50 for each drug. Bar plot showing number of drugs for which the expression vs IC50 correlation coefficient was among the top 50 SCCs. FAM129B predicted resistance for the largest number of drugs. **E** Bar plot showing number of drugs for each of the 36 genes identified from the GDSC database is significantly (adjusted *P* < 0.05) associated with high SCC (gene expression vs. IC50) in the CTRP database.

To systematically discover the biomarkers that are predictive of anti-cancer drug response we followed the computational framework shown in Fig. 1A. The GDSC database includes IC50 of 192 drugs on 809 cancer cell lines, while the COSMIC database provides the microarray-based Z-score expression profile of approximately 16,000 genes in 970 cancer cell lines. 777 cancer cell lines were common between these databases. Higher IC50 values indicate greater resistance. We hypothesized that if high expression of a gene is correlated positively with drug response (IC50) then the gene could be associated with drug resistance. In contrast, if high expression of a gene is negatively correlated with drug response, then the expression of the gene could be a marker of sensitivity to the drug.

### Pairwise correlation analysis identifies NIBAN2/FAM129B as a candidate marker for chemoresistance in cancer cell lines

We demonstrate the results in pan-cancer for one of the top genes whose expression showed among the highest correlation with IC50 of several drugs, FAM129B. Temozolomide (DNA alkylating agent), Cytarabine (Cytidine antimetabolite) and Gemcitabine (Cytidine antimetabolite) all lead to DNA damage and halt DNA replication. The plots of IC50 for the three drugs versus expression of FAM129B, in all 777 cancer cell lines are shown along with the correlation coefficients and the p-value (Fig. 1B). These correlation values for 16,248 genes were ranked from highest to lowest. FAM129B ranked as the best correlated gene (adjusted *P* = 7.66e−47) for Temozolomide resistance (Supplementary Fig. 1A). In addition, FAM129B ranked 7 (adjusted *P* = 3.96e−34) and 13 (adjusted *P* = 3.97e−24) for resistance against Cytarabine and Gemcitabine, respectively. To validate this finding, we performed correlation analysis of expression of 18,541 genes with drug sensitivity of Temozolomide, Cytarabine and Gemcitabine in the CTRP dataset. The correlation of FAM129B expression with IC50 of the drugs was consistently within the top 2% of the genes (Supplementary Figure 1B). Although in the CTRP dataset, FAM129B ranked lower for Temozolomide and Gemcitabine, there were other drugs where it ranked higher, e.g. it was the third candidate for Sorafenib resistance (rho = 0.28, adjusted *P* = 6.53e−12).

### Identification of a set of genes predictive of drug resistance

To identify genes most predictive of resistance to multiple drugs, we followed the strategy described in the schematic Fig. 1C. For each of 51 FDA approved drugs we ranked the correlation values of each gene (from GDSC) giving the lowest rank to the highest correlation value. We kept genes which were significantly correlated (adjusted *P* < 0.05). For each drug we hypothesized that the genes which had the 50 highest correlation values with IC50 (out of ~16,000) are predicting resistance to that drug. To identify genes that are predictive of resistance to a large number of drugs, we focused on 36 genes that were predictive of resistance to 20 or more drugs (Fig. 1D). FAM129B predicted resistance to the highest number of drugs (39 of the 51 FDA approved drugs), with diverse mechanism of actions which included alkylating agents, anti-inflammatory agents, anti-metabolites, kinase inhibitors, PARP inhibitors, NAE inhibitors, anti-estrogens, BCL-2 inhibitors, cytoskeleton inhibitors, topoisomerase inhibitors, mTOR inhibitors, HDAC inhibitors and proteosome inhibitors (as shown in Supplementary Data 1). Experimentally, silencing of FAM129B has been shown to produce sensitivity to Oxaliplatin in breast cancer cells^23^.

We next validated this finding in the independent CTRP dataset, where there were 34 FDA approved drugs in common with the GDSC. We repeated the workflow and performed correlation analysis of 18,541 gene expression with AUC values of 34 drugs. The genes were ranked from the most correlated to the least correlated. Although the rank of a gene for a particular drug is well correlated between GDSC and CTRP, the exact ranks in GDSC and CTRP are randomly scattered around zero, as can be seen in the residual plots (Supplementary Fig. 2). Thus, despite the general correlation, the exact rank of a gene in predicting resistance to a drug varies between the two databases and we decided against using concordance of rank as the method to pick the list of predictive genes. Instead, we simply selected the 36 drug-resistance genes in GDSC and tested whether these 36 genes predicted resistance to multiple drugs in CTRP. The 36 genes predictive of resistance in ≥20 drug in GDSC were included in the top 2% of the genes that were significantly (adjusted *P* < 0.05) correlated with multi-drug resistance in the CTRP data (Fig. 1E). FAM129B showed resistance for 17 of the 34 drugs and was among the best predictors of resistance in the CTRP dataset.

### Identifying drug resistance of cancer cell lines based on a polygenic score of 36 genes

**Fig. 2:**
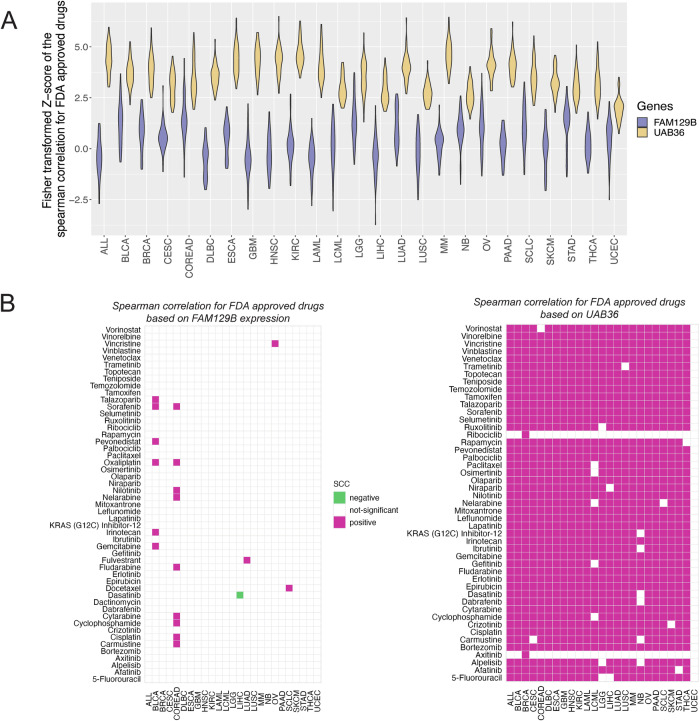
Correlation of IC50 with UAB36 score is higher than with FAM129B expression alone across cancer cell lineages and against different drugs. **A** Violin plot shows the distribution of Fisher transformed z-score obtained from the spearman correlation of IC50s for FDA approved drugs with expression of FAM129B (colored in purple) and with UAB36 (colored in yellow). In every cancer type, the violin plot for UAB36 was significantly different from that for FAM129B (*p* < 10^−12^ in Wilcoxon Rank Sum test). **B** Heatmap shows spearman correlation of IC50s of FDA-approved drugs in specific lineages versus expression of FAM129B or UAB36 score. Pink box: Positive SCC and FDR < 0.05. Green box: Negative SCC and FDR < 0.05. White box: none of the above. The analyses are shown from the GDSC dataset.

Once we had identified 36 genes that predicted resistance to a substantial number of drugs, we wondered whether a polygenic score specifically derived from these 36 genes, UAB36, has a higher correlation with the IC50 to a specific drug than that of the best gene, FAM129B (see Methods). We calculated the SCCs of UAB36 vs. IC50 (of all drugs, on all cell lines) and polygenic score vs. IC50 (of all drugs on all cell lines). The distribution of the raw correlation coefficient values and their conversion to Z-score using Fisher’s Z-transformation is described in methods and is shown in Supplementary Figure 3. The distribution of correlation coefficients (expressed as Z-score) of UAB36 score with IC50 is clearly much higher than that of FAM129B with IC50s (*p* < 10^−12^ in Wilcoxon Rank Sum test) in all cell lineages (Fig. 2A).

We also performed the analysis after separating the drugs in different lineages and determining the correlation coefficient of UAB36 score with the IC50 of any drug. After correcting for FDR, the UAB36 score showed a significantly higher correlation with the IC50 than FAM129B’s correlation with IC50, both in terms of cell-lineage and drug used (Fig. 2B). The spearman correlation coefficient values and FDR corrected *p* values of FAM129B with IC50 of drug are provided in the Supplementary Data 2. Similarly, the values for UAB36 are provided in the Supplementary Data 3. Because we selected the genes for the polygenic score using IC50 of multiple drugs in GDSC, we expected UAB36 based polygenic scores to be effective against different drugs in different lineages even in CTRP. Indeed, in the CTRP database, UAB36 showed significant positive correlation with 5-Fluorouracil in the large intestine (rho: 0.5; *p* value: 0.0003726), Crizotinib in the lung cancer (rho: 0.19; *p* value: 0.02585), Axitinib in kidney cancer (rho: 0.71; *p* value: 0.0008528) and Gemcitabine in the ovarian cancer (rho: 0.67; *p* value: 8.22e−05) as shown in Supplementary Fig. 4.

### UAB36 outperforms established gene signatures in predicting tamoxifen resistance in breast cancer cells

**Fig. 3:**
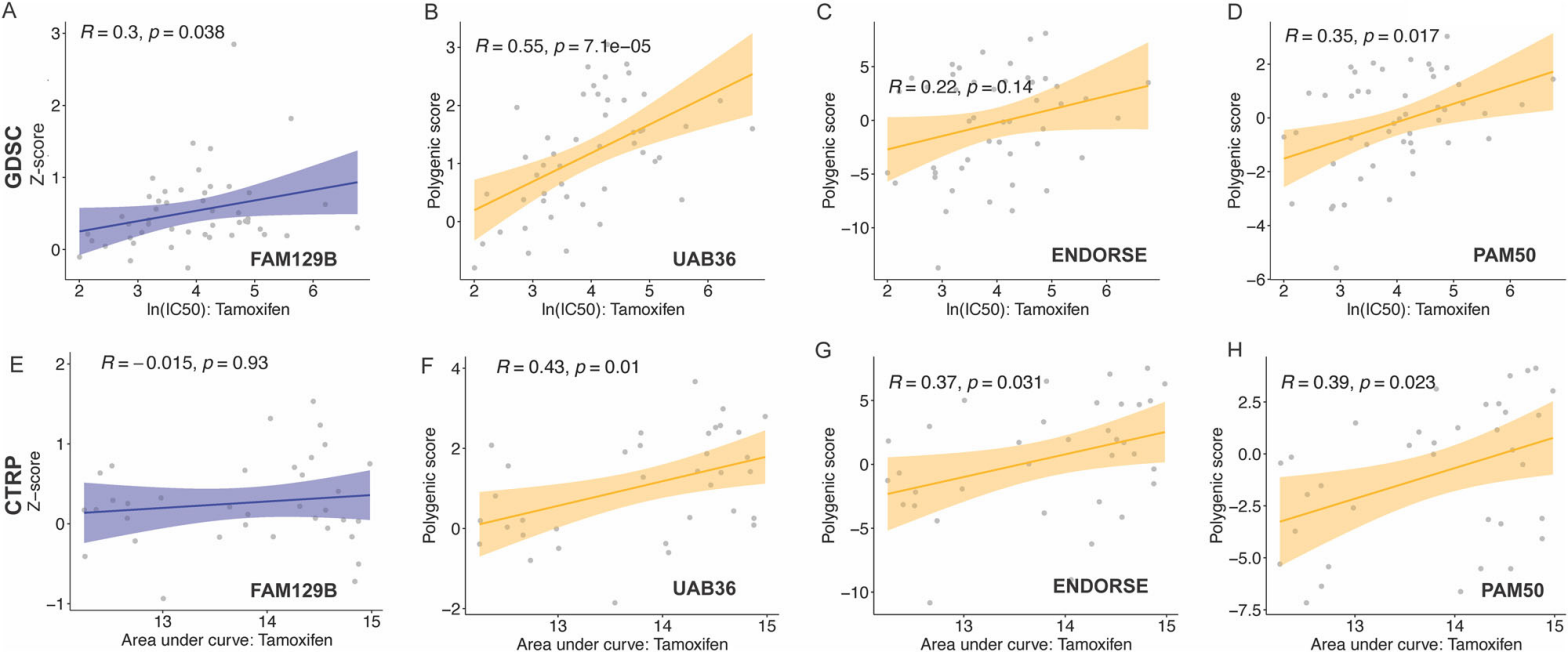
UAB36 is better correlated with IC50 of Tamoxifen in breast cancer cells compared to FAM129B, ENDORSE or PAM50. **A**–**D** Breast cancer cell lines (*n* = 48) from the GDSC database**. E**–**H** Breast cancer cell lines (*n* = 35) from the CTRP database. Scatter plot shows spearman correlation of IC50 (or AUC) to Tamoxifen with expression level of FAM129B, Polygenic score of UAB36 gene set, Polygenic score of ENDORSE gene set and Polygenic score of PAM50 gene set, as indicated. The IC50 values from the GDSC database are expressed in micromolar concentrations. The AUC values from the CTRP database range from 0 to 29.4.

Turning to a specific cancer, the analysis in Fig. 2B showed that in the GDSC dataset breast cancer cells the UAB36 score had a very high correlation to IC50 to tamoxifen. In support of this, the UAB36 score was significantly (*P* = 8.63e−03) higher in tamoxifen resistant subclones of MCF7 cell lines than in tamoxifen sensitive subclones from the GSE26459^24^ (Supplementary Fig. 5). The UAB36 score is highly correlated with IC50 of tamoxifen (rho = 0.55, *P* = 7.1e−05) than FAM129B expression alone with the IC50 (rho = 0.3, *P* = 3.80e−02), in 48 breast cancer cell lines (Fig. 3A, B). The polygenic score was next tested in the breast cancer cells (*n* = 35) from the CTRP dataset. We could not include KRT8 gene expression into the model as its expression was not reported in the CTRP dataset. With the same polygenic score formula, the score obtained from the 35 genes remained significantly associated with tamoxifen resistance in this independent CTRP dataset and here again, the polygenic score is better correlated with IC50 than FAM129B alone (Fig. 3E, F).

To assess the efficacy of UAB36 in comparison to published gene signatures in breast cancer cells, we downloaded the following gene sets: ENDORSE which includes 63 genes linked to ER^+^ breast cancer patient survival treated with endocrine therapy^25^, and PAM50, a widely accepted classifier consisting of 50 genes for molecular subtyping of breast cancer. A study by Chia et al.^26^ showed in NCIC CTG MA.12 clinical trial, where premenopausal women with primary breast cancer treated with adjuvant tamoxifen, PAM50 subtyping could predict response to tamoxifen treatment. We then calculated the spearman correlation of the expression of these genes with the IC50 of tamoxifen in the breast cancer cells from the GDSC and applied the same polygenic score formula to create the polygenic score for ENDORSE genes and PAM50 genes. Intriguingly, the polygenic scores derived from these sets exhibited lower correlation strengths, as depicted in Fig. 3C, D, when compared with the UAB36 score (Fig. 3B).

Similarly, we created the polygenic score based on gene sets in the CTRP, and here again UAB36 demonstrated a stronger positive correlation with the IC50 of tamoxifen than the ENDORSE or PAM50 gene sets (Fig. 3F–H).

No genes from UAB36 overlapped with those in ENDORSE and PAM50 sets.

### Enrichment of tamoxifen resistance gene sets in breast cancer patients with a high UAB36 score

With the promising results observed in the breast cancer cells, we next asked in breast cancer patients whether high UAB36 score was correlated with the expression of genes known to be associated with tamoxifen resistance, a surrogate marker of such resistance.

**Fig. 4:**
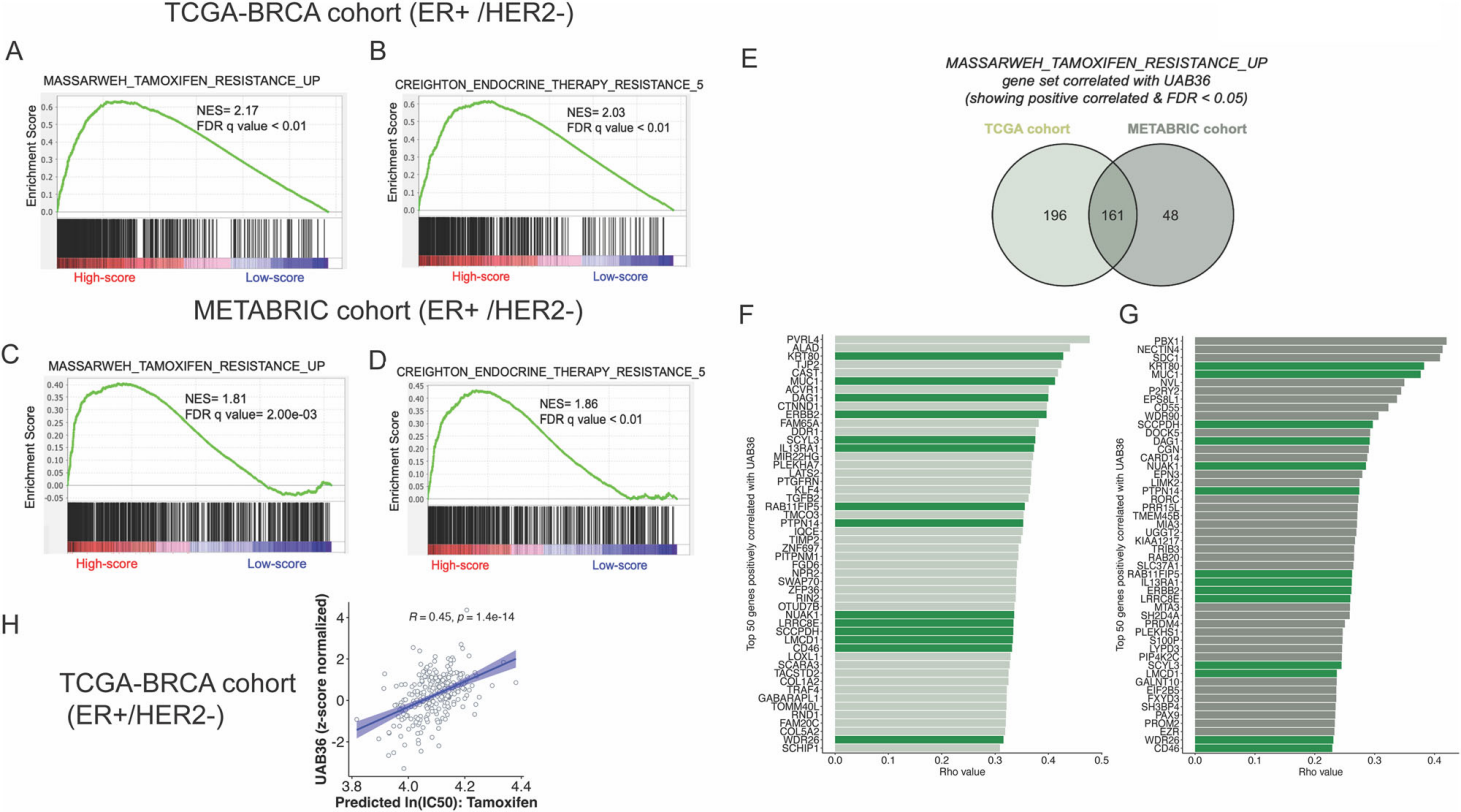
Tumors with high UAB36 score are enriched for Tamoxifen resistance genes in breast cancer (ER+/HER2−) patients. Enrichment plot shows enrichment of MASSARWEH_TAMOXIFEN_RESISTANCE_UP gene set (**A**) and CREIGHTON_ENDOCRINE_THERAPY_RESISTANCE_5 gene set (**B**) in high UAB36 expressing tumors from the TCGA-BRCA (ER+/HER2−) cohort. The TPM expression values of each gene is normalized to z-scores across samples. NES normalized enrichment score, FDR false discovery rate. **C**, **D** Same as **A**, **B** except in the high UAB36 expressing tumors from the METABRIC breast cancer (ER+/HER2−) cohort. **E** Venn diagram showing the overlap of MASSARWEH_TAMOXIFEN_RESISTANCE_UP gene set which are significantly (FDR < 0.05) positively correlated with UAB36 between the TCGA-BRCA (ER+/HER2−) cohort and METABRIC breast cancer (ER+/HER2−) cohort. **F** Bar plot showing the spearman correlation coefficients of the top 50 MASSARWEH_TAMOXIFEN_RESISTANCE_UP genes with significant positive correlation with UAB36 score in the TCGA-BRCA (ER+/HER2−) cohort. **G** Same as **F** except in the METABRIC (ER+/HER2−) cohort. Here, bars colored in dark green are the genes which are in common between TCGA and METABRIC cohort. **H** Scatter plot showing positive correlation of UAB36 score with the IC50 for tamoxifen in the TCGA-BRCA (ER+/HER2−) cohort predicted by Li et al.^16^.

We performed GSEA on z-score normalized RNA-Seq data from patient tumors in the TCGA breast cancer (TCGA-BRCA), specifically focusing on ER^+^/HER2^−^ tumors. We used the same polygenic score formula from the 36 genes, using the correlation coefficients with IC50 for tamoxifen on the breast cancer cell lines in the GDSC database and the expression levels of the genes in the tumors. Subsequently, patients were stratified into risk groups based on q1 and q4 quartile cut-off. We found a significant enrichment of MASSARWEH_TAMOXIFEN_RESISTANCE_UP gene set (NES: 2.17 and FDR *q* value < 0.01) in the high UAB36 score group (q4 cut-off) as shown in Fig. 4A. Upregulation of these genes was associated with resistance to tamoxifen in MCF-7 mouse xenografts^20^. A similar result was obtained for CREIGHTON_ENDOCRINE_THERAPY_RESISTANCE_5 gene set (NES: 2.03 and FDR q value < 0.01; Fig. 4B), whose expression is associated with resistance to endocrine therapy in MCF-7 mouse xenografts expressing ESR1^21^. The robustness of these associations was validated in an independent METABRIC cohort of ER^+^/HER2^-^ breast cancer tumors that received adjuvant endocrine therapy^27,28^. Consistent with the TCGA cohort, the high-UAB36-score group demonstrated significant enrichment for the same two gene sets with NES 1.81 and 1.86, respectively (Fig. 4C, D).

The GSEA suggest that the UAB36 polygenic score should be correlated with genes known to be involved in tamoxifen resistance. We calculated the spearman correlation of expression of each of the genes in the MASSARWEH_TAMOXIFEN_RESISTANCE_UP gene set (*n* = 582) with the UAB36 score in the ER^+^/HER2^-^ TCGA-BRCA tumors. 357 of the 576 genes were significantly (FDR < 0.05) positively correlated with UAB36. A parallel analysis within the ER^+^/HER2^-^ METABRIC tumors showed that 209 of the 561 genes were significantly positively correlated with UAB36 score. 40% of the genes significantly positively correlated with UAB36 score in the TCGA cohort were also similarly correlated in the METABRIC cohort (Fig. 4E). We extracted the top 50 positively correlated genes from both the cohorts (Fig. 4F, G) and identified 14 genes that were in common between the two cohorts within the top 50 gene lists, highlighting a subset of tamoxifen resistance associated genes that are most strongly and reproducibly correlated with UAB36 score. Thus, there is indeed a significant and conserved correlation of genes known to be associated with tamoxifen resistance with the UAB36 score. In addition, the high UAB36 score group of patients from the TCGA showed enrichment of expression of gene sets associated with multiple drug resistance, gefitinib resistance, fluorouracil resistance, response to cisplatin and response to oxidative stress as shown in Supplementary Fig. 6. The UAB36 genes themselves are enriched in genes involved in cellular response to stress and several cancer survival pathways as shown in Supplementary Table 2.

There have been previous attempts to predict resistance to drugs using gene expression datasets. For example, Li et al.^16^ used machine learning approaches on GDSC cancer cell lines to predict IC50 values of 272 drugs for TCGA tumors. We downloaded the predicted IC50 values of tamoxifen for TCGA-BRCA cohort https://manticore.niehs.nih.gov/cancerRxTissue/ and correlated these predicted IC50 values of the tumors with the UAB36 scores of the same tumors. The predicted IC50s to tamoxifen are positively correlated (rho = 0.45, *P* = 1.4e−14) with UAB36 score (Fig. 4H), again suggesting that the UAB36 score performs well to predict resistance/sensitivity to tamoxifen in patient tumors.

### UAB36 predicts poor outcome in ER^+^/HER2^-^ breast cancer patient treated with tamoxifen

**Fig. 5:**
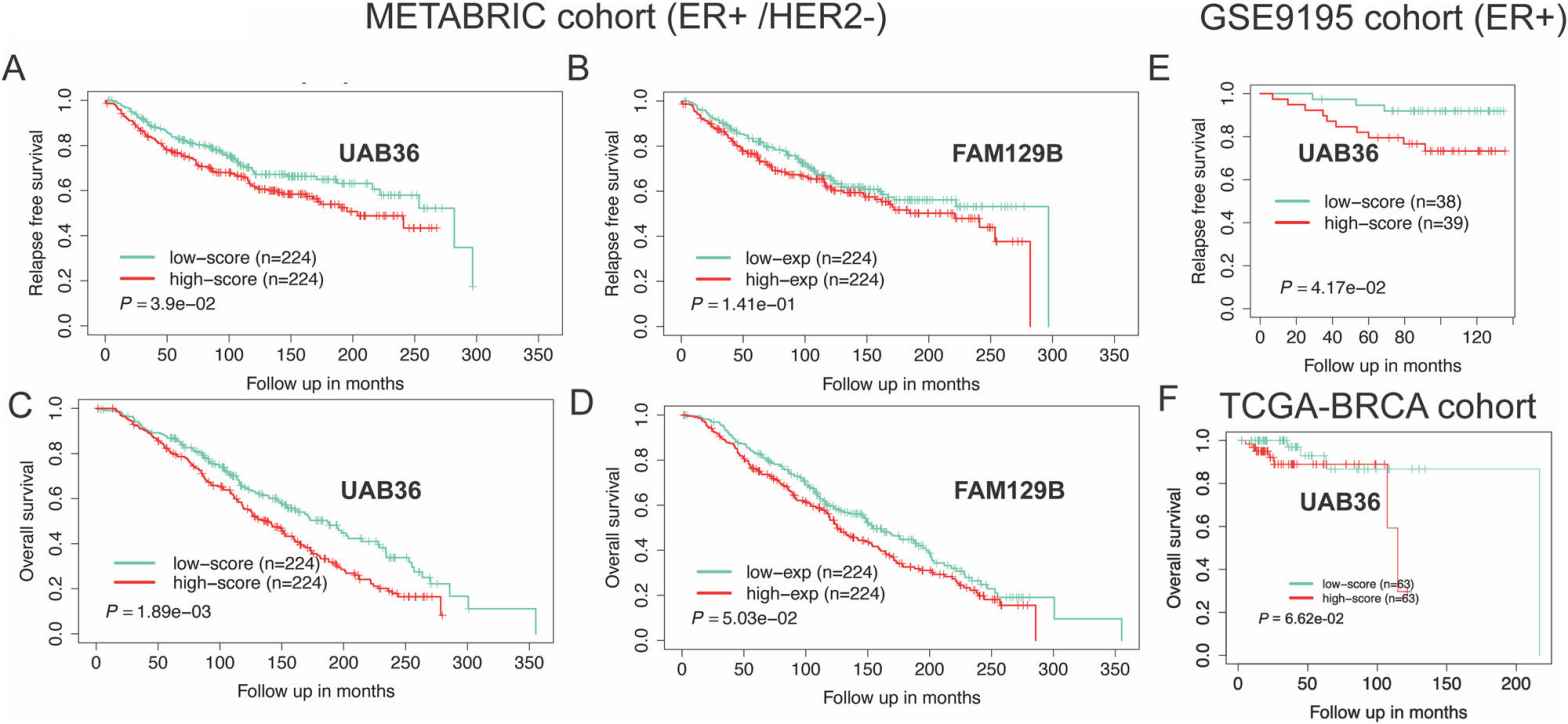
UAB36 is strongly associated with poor outcome in breast cancer (ER+/HER2−) patients treated with Tamoxifen. **A**–**D** In the METABRIC (ER+/HER2−) patient tumors treated with tamoxifen. **A** Relapse free survival Kaplan–Meier curves of tumors stratified using UAB36. **B** Relapse free survival Kaplan–Meier curve of tumors stratified using expression of FAM129B. **C** Overall survival Kaplan–Meier curves of tumors stratified using UAB36. **D** Overall survival Kaplan–Meier curve of tumors stratified using expression of FAM129B. Patients were stratified into two groups based on quartiles (Q1 and Q4) threshold. The *P* values for the survival analysis were obtained using a Mantel log-rank test (two-sided). **E** In the GSE9195 breast cancer (ER+) patients treated with tamoxifen. Relapse free survival Kaplan–Meier curves of tumors stratified using UAB36. Patients were stratified into two groups based on median threshold. **F** In the TCGA-BRCA cohort treated with tamoxifen. Overall survival Kaplan–Meier curves of tumors stratified using UAB36. Patients were stratified into two groups based on quartiles (Q1 and Q4) threshold.

If a high UAB36 score predicts high IC50 of breast cancer cells to tamoxifen, we should expect that high UAB36 score should predict poor outcome in breast cancer patients treated with tamoxifen. To elucidate the prognostic value of UAB36 on ER^+^ cancers treated with tamoxifen, we performed Kaplan–Meier survival analysis in the 448 patients of the METABRIC cohort. Compared with tumors in the low UAB36-score group, those with a high-score were significantly associated with tumor recurrence and patient death (Fig. 5A–C). It is noteworthy, that the separation between two curves in Fig. 5A–C is wider than with FAM129B alone (Fig. 5B–D) for relapse free survival and overall survival, supporting that the polygenic drug resistance risk score derived from 36 genes is better than FAM129B alone for predicting outcome post-tamoxifen treatment.

Similarly, we tested prognostic association of UAB36 in an independent cohort GSE9195^29,30^, where 77 ER^+^ breast cancer patients were treated with tamoxifen in the adjuvant setting. Using the same UAB36 score formula, the high-score group correlated with tumor relapse (Fig. 5E). Due to a smaller sample size of this cohort, we employed a median cut-off for risk stratification. Lastly, we examined the 252 TCGA-BRCA patients for which we have information about outcome following treatment with tamoxifen^31^. Using a quartile-based stratification, patients with high UAB36 scores were again found to have worse survival outcomes, though the level of significance was marginal (Fig. 5F). Thus, in three different cohorts, METABRIC, GSE9195 and TCGA-BRCA, a high UAB36 score was associated with poor outcome.

### UAB36 outperforms the prognostic ability of ENDORSE and PAM50 and is a predictor of outcome independent of known clinical risk factors in breast cancer patients

To evaluate the prognostic potential of UAB36 against the established gene signatures ENDORSE and PAM50, we performed Cox regression analysis for overall survival in the METABRIC ER^+^/HER2^-^ breast cancer cohort treated with tamoxifen. Additionally, the impact of clinical risk factors, including age, tumor stage and tumor grade, on patient survival was assessed. As depicted in Table 1, we first stratify the patients into two groups based on median threshold of UAB36. In the univariate analyses, patients with high UAB36 score exhibited significant hazard ratio (HR) of 1.33 (*p*-value: 1.11e−03), indicating patients with high UAB36 score have a higher risk of death after tamoxifen treatment compared to those with a low UAB36 score. In comparison a high score in the ENDORSE or PAM50 gene sets were also associated with increased risk of poor outcome as demonstrated by significant higher hazard ratio. Age and patient tumor grade and tumor stage showed significant association with poor survival, as expected from the Literature^25,32,33^.

**Table 1:** Cox regression analysis of UAB36 with established gene signatures and clinical risk factors for overall survival in the tamoxifen treated METABRIC ER+/HER2− cohort (*n* = 895)

	Univariate model		Multivariate model	
Variables	HR	*P*	HR	*P*
UAB36 *(high vs. low)*^*a*^	**1.33**	**1.11E−03**	**1.28**	**2.34E−02**
ENDORSE *(high vs. low)*^*a*^	**1.26**	**7.93E−03**	1.02	9.22E−01
PAM50 *(high vs. low)*^*a*^	**1.39**	**1.63E−04**	1.05	7.58E−01
AGE *(continuous)*	**1.06**	**<2E−16**	**1.06**	**<2E−16**
TUMOR STAGE	**1.69**	**8.40E−09**	**1.39**	**7.66E−04**
TUMOR GRADE	**1.18**	**1.63E−02**	1.12	2.09E−01

^a^In both univariate and multivariate Cox regression analysis, the gene set median drug score was used as a cutoff to divide patients into high and low score groups.

*P-value* in bold typeface indicates statistical significance (*P* < 0.05).

To account for potential confounding factors, we then performed the multivariate Cox regression analysis using the three polygenic scores (together) along with the three clinical co-variates (age, grade and stage). The association between UAB36 and patient survival remained significant (HR = 1.28, *p*-value = 2.34E−02) even after adjusting for the clinical co- variates and the other polygenic scores. In contrast, ENDORSE and PAM50 lost their significance in predicting patient survival outcome when the clinical co-variates were included with (Table 1) or without (Supplementary Table 3) taking UAB36 into consideration. Age remained a significant factor in the model, but with a small effect size, but tumor stage maintained its association with poor survival. Tumor grade lost its significance in the multivariate model.

A similar multivariate Cox model in another dataset, GSE9195, showed that high UAB36 score was significant and an independent predictor of patient tumor relapse even with age and tumor grade as co-variates (Table 2).

**Table 2:** Cox regression analysis of UAB36 with established clinical risk factors for relapse free survival in the tamoxifen treated GSE9195 ER+ cohort (*n* = 77)

	Multivariate model	
Variables	HR	*P*
UAB36 *(high vs. low)*^*a*^	**3.9**	**4.38E−02**
AGE *(continuous)*	1.02	5.29E−01
TUMOR GRADE	2.37	5.35E−02

^a^In multivariate Cox regression analysis, the gene set median drug score was used as a cutoff to divide patients into high and low score groups.

*P-value* in bold typeface indicates statistical significance (*P* < 0.05).

These results collectively indicate UAB36 polygenic score holds promise as a therapeutic response biomarker and an independent predictor of drug resistance risk and poor survival in ER^+^/HER2^-^ breast cancer patients undergoing tamoxifen treatment.

### All genes in UAB36 are required in the UAB36

To confirm that a set of 36 genes is essential for the UAB36 gene signature, we constructed a competing 35-gene signatures (UAB36-minus-1) by deleting one gene in turn from the set and asked whether systematic removal of a gene altered the ability to predict patient death probability, as measured by increase or decrease of HR. In the METABRIC cohort, we performed multivariate Cox regression analysis for each of the “36-minus-1” gene signatures, taking ENDORSE, PAM50 gene signatures as well as age, tumor grade and tumor stage as confounding factor, and compared the hazard ratio with the original hazard ratio of UAB36 (HR = 1.28). Removal of most of the genes, decreased HR, thus decreasing predictive ability. However, single removal of a small number of genes, ABCC3, ADAM9, ANXA2, ARHGAP29, CCN1, EPHA2, GIPC1, GNG12, KIAA152, LMNA, MYOF or S100A10 gene, significantly increased the HR (Supplementary Data 4). Similarly, we repeated the above approach of “UAB36-1” in the GSE9195 cohort. Here, we found removal of either TM4SF1, MET, GNG12, TNFRSF12A, CAPN2, and TUFT1 significantly increased the HR (Supplementary Data 5). Between the two cohorts GNG12 was the only gene whose removal consistently increased the HR in both cohorts, as shown in Table 3.

**Table 3:** Systematic removal of genes from the UAB36

	METABRIC		GSE9195	
Variables	HR	*P*	HR	*P*
UAB36	1.28	2.34E−02	3.92	4.38E−02
UAB36-1 *(GNG12)*	1.32	9.94E−03	4.75	2.52E−02
UAB36-2*(DCBLD2, TM4SF1)*	1.31	1.15E−02	4	4.02E−02
UAB36-2*(ARHGAP29, TM4SF1)*	1.32	9.56E−03	4.8	2.19E−02
UAB36-3*(FAM129B, ANXA2, S100A10)*	1.39	2.61E−03	4.4	3.04E−02

Multivariate Cox regression of UAB36 with established gene signatures and clinical risk factors in the METABRIC cohort and GSE9195 cohort.

Next, we prepared 36 competing 34-gene signatures, i.e. “UAB36-minus-2”, by deleting any two genes from the set. Removal of two sets, DCBLD2 + TM4SF1 or ARHGAP29 + TM4SF1 increased the HR in both METABRIC and GSE9195.

Further, we proceeded with 33-gene signatures “UAB36-3” and found that removal of a set containing FAM129B, ANXA2 and S100A10 increased the HR.

Although the removal of selected 1, 2 or 3 genes increased the HR (Table 3), the increase was minor in one cohort or the other. Therefore, for the current study, we decided to keep all genes in UAB36 as essential. In the future, as new studies publish new breast cancer cohorts treated with tamoxifen, we will test if removal of these genes from UAB36 significantly improves the HR by an even larger extent.

## Discussion

Despite advances in targeted agents and systemic chemotherapies, the majority of patients develop resistance to these therapies and eventually relapse. In this study, we examined the relationship between drug response (IC50 or AUC) of chemotherapy agents and gene expression levels in cancer cell lines. The large-scale drug screens in GDSC and CTRP generated invaluable sensitivity data for hundreds of drugs measured across approximately 1000 of cancer cell lines. Pairwise spearman’s correlation analysis between drug response and whole gene expression patterns across cancer cell lines identified genes whose expression was associated with drug resistance or sensitivity. This approach led us to identify FAM129B and an additional 35 genes whose expression levels were statistically significantly associated with IC50 of multiple drugs across a pan-cancer set of cell lines in the GDSC dataset and validated in the CTRP dataset.

One mechanism by which these genes might predict higher resistance to drugs will now be discussed. FAM129B also known as NIBAN2, is a protein-coding gene and its elevation produces platinum resistance. Cheng et al.^23^ showed that FAM129B, by interfering with the interaction of the Cul3 substrate adapter, Kelch1, with the substrate Nrf2, elevates Nrf2 protein, suppresses reactive oxygen products and thus make breast cancer cells and colorectal cancer cells more resistant to Oxaliplatin. Our results confirm this finding, with significant positive correlation of FAM129B with IC50 to Oxaliplatin in colorectal cancer (rho = 0.3; adjusted *P* = 3.13e−02) from the GDSC database. In addition, as Nrf2 is a transcription factor and a master regulator of antioxidative stress, we hypothesized that in cancers with elevated FAM129B, the expression of Nrf2 and its targets increase. This would make cancer cells resistant to drug treatment by enabling them to better tolerate stress from Reactive oxygen species (ROS). FAM129B mRNA showed significantly positively correlated with Nrf2 targets in several TCGA cancers as shown below in Supplementary Fig. 7. In Supplementary Fig. 6 and Supplementary Table 2, we show that the UAB36 genes are also enriched in genes involved in response to cellular stress and are associated with increased expression of genes involved in drug resistance and oxidative stress response pathways. Thus, we hypothesize that the success of UAB36 in predicting higher IC50 to many different drugs is because the expression of these genes is associated with activation of cellular stress response pathways. For FAM129B we know the biochemical pathway by which cellular stress response pathways are activated, but clearly more experimental work is necessary to determine how the remaining 35 genes are correlated with cellular stress response.

In addition to direct involvement in cellular response pathways, UAB36 may be predictive of drug resistance through indirect mechanisms. For example, tumors with a high UAB36 score were positively correlated with expression of PBX1, MUC1, KRT80 and ERBB2. Magnani et al.^34^ demonstrated PBX1 as a pioneer factor in ER^+^ breast cancer. PBX1 is required in EGF signaling in the ER^+^ breast cancer cells, as silencing of PBX1 in MCF-7 cells slowed the EGF induced cell proliferation. Thus, by promoting EGF signaling, PBX1 could help breast cancer cells survive estrogen receptor blockade. Indeed, they showed high expression of PBX1 is associated with poor survival in ER^+^ breast cancer patients. Pitroda et al.^35^ showed overexpression of MUC1 leads to differential expression of genes involved in cholesterol and fatty acid metabolism. This MUC1-induced lipid metabolism gene signature showed poor survival of tamoxifen treated breast cancer patients. Perone et al.^36^ showed in an aromatase inhibitor-ER^+^ breast cancer cells, epigenetic reprogramming leads to upregulation of KRT80, driven by the activation of new enhancers by SREBP1. This upregulation of KRT80 promotes tumor stiffness and cancer invasion. Kurokawa et al.^37^ demonstrated overexpression of ERBB2/HER2 in MCF-7 breast cancer cells leads to tamoxifen resistance through the activation of MAPK activity. Thus, correlation of UAB36 score with the expression of genes that promote tamoxifen resistance is another explanation for the success of this score in predicting tamoxifen resistance.

As mentioned in the introduction, we set out to test whether a cell line derived polygenic resistance marker will predict poor outcome in response to specific drug therapy in a specific cancer. We focused on the treatment of breast cancer with Tamoxifen (brand names: Nolvadex, Soltamox), which is FDA approved to treat hormone-receptor positive and early-stage breast cancer patient after chemotherapy and surgery. Tamoxifen is a selective estrogen modulator that competes with estrogen for estrogen receptor binding inhibiting estrogen function in breast cancer progression. Studies have shown that in breast cancer patients who were treated with tamoxifen for 5 years, the risk of tumor recurrence elsewhere in the body is 26% after 20 years^38^. In another study, the rate of tumor recurrence is more than 10% during first five years, suggesting the frequent occurrence of resistance to the drug^39^. In addition, 20-30% breast cancer tumors were resistant to tamoxifen therapy^40^.

The polygenic UAB36 score constructed from the correlation coefficient of transcriptomics in breast cancer cell lines using patient tumor gene expression was effective at predicting tamoxifen response in ER^+^/HER2^-^breast cancer patients. The correlation of the UAB36 score in tumors with genes that are known to be associated with tamoxifen resistance was encouraging and showed that a score derived from cell-line transcriptomics could correlate with the resistance to the drug in breast tumors.

Consistent with this, the UAB36 score predicts poor outcome after tamoxifen treatment in the ER^+^/HER2^-^ breast cancer patients from the METABRIC, GSE9195 and TCGA-BRCA cohorts. We are very encouraged by the observation that the polygenic UAB36 score not only performs better than the best single gene, FAM129B, but also performs better than two existing polygenic scores, ENDORSE and PAM50, for predicting patient survival after tamoxifen treatment. In addition, UAB36 score was unique amongst the other two polygenic scores in being a predictor of cancer outcome independent of known clinical co-variates (age, tumor grade and tumor stage) known to influence outcome in breast cancer. Thus, the UAB36 score can be used to stratify patients into high-score and low-score groups for tamoxifen treatment to guide personalized therapy decisions.

As mentioned above, there have been other attempts to use cell line transcriptomics to predict drug resistance or sensitivity. Shee et al.^15^ using correlation analysis of transcriptomes and IC50 of drugs from GDSC, CTRP and NCI identified SLFN11 gene expression as a marker for drug sensitivity across solid cancers. Li et al.^16^, applied machine learning approach to identify a set of genes that can accurately predict the sensitivity of a particular drug in cancer cells and to predict IC50 of a drug for TCGA tumor. Jang et al.^17^, developed a web platform which uses drug response data from CTRP and gene expression of cancer cell lines from CCLE and provides resistant gene signatures which are differentially expressed in resistant cancer cell groups. Zhang et al.^41,42^ developed a computational framework using the in vitro drug screens to *predict* drug responses in castration resistance prostate cancer cell lines. However, unlike what we have done here with UAB36, these studies did not test whether their sets of genes are useful for predicting outcome in patients subjected to a specific treatment.

Genes in UAB36 were identified because they had the highest correlations (FDR < 0.05) with IC50s of >20 drugs in GDSC. As a result, the polygenic UAB36 score predicted resistance to multiple drugs in multiple cancer cell lineages (Fig. 2B). After selecting the genes, the only modification we made to the formula for applying to TCGA breast cancer patients treated with tamoxifen, was to use the SCC of the UAB36 gene mRNA levels to the IC50 of the specific drug (in this case, tamoxifen) specifically in breast cancer cell lines. We will in the future examine whether this approach can be extended to predict patient outcomes in response to other drugs in other tumors (e.g. temozolomide on glioblastomas and lower grade gliomas) using the same UAB36 genes.

We tested whether our approach could identify markers which are predictive of drug sensitivity. The correlation analysis of the whole transcriptome to the IC50 (or AUC) of tamoxifen in breast cancer cells identified 369 genes which are significantly negatively correlated from the GDSC database, and 375 genes from the CTRP database. Only 5 genes were common between GDSC and CTRP such as DEPDC5, ACTL10, SOBP, SLC38A11 and MANBA. A polygenic score derived from these 5 genes was negatively correlated with tamoxifen IC50 in GDSC and tamoxifen AUC in CTRP (Supplementary Fig. 8). However, when we used a polygenic score from these studies to predict patient outcome in the METABRIC cohort, the score failed to be predictive. Because of the lack of this utility in prediction of clinical outcome, we have to improve our method to identify sensitivity markers that will be useful for patient management.

The ability to predict cancer patient drug sensitivity and outcome with a score whose formula is derived from the response of an independent set of cancer cells to drugs in vitro is exciting and provides support for extending such studies to multiple different cancers.

Decreased expression of Tumor suppressor genes (TSGs) such as p53 is associated with drug resistance^43^. Although increased expression of the UAB36 genes is associated with drug resistance, we examined whether UAB36 was selectively enriched or dis-enriched for TSGs^44^. TNFRSF12A, EPHA2, YAP1, GPRC5A, PLK2 and ARHGAP29 are known TSGs in the UAB36 set. Fisher’s exact test shows that TSGs were enriched in the UAB36 compared to genes not in UAB36 (Odd’s ratio = 2.48; *p*-value = 0.0491). As a negative control, we examined the 28 genes with lowest correlation value with IC50 for at least 20 drugs, that we tested for prediction of sensitivity of drug. In this group TSGs were depleted, however the result was not significant (Odd’s ratio = 0.46; *p*-value = 0.7196). The enrichment of TSGs among the UAB36 genes that predict resistance is paradoxical because the expectation is that loss of TSGs make the cancer cells more aggressive. Indeed, a polygenic score made of these 6 genes, TSG6, had a lower and non-significant correlation with IC50 of Tamoxifen (rho = 0.24; *p*-value = 0.1032) compared to UAB36. A similar analysis for low expressing genes (1625 genes whose mean expression level in the entire GDSC cancer cell line group was below the 10^th^ percentile) showed no overlap of low expression genes in the UAB36 set.

In summary, a polygenic score built from FAM129B, and 35 other genes associated with high IC50 in vitro can be used as a biomarker for predicting poor outcome in patients in three different cohorts and is an independent predictor of outcome even after taking into consideration available clinical co-variates. In breast cancer this polygenic score is better than a single gene alone for stratifying ER^+^/HER2^-^ patients into those that have better survival (disease-free and overall) after tamoxifen treatment and those that do not. The UAB36 score is correlated with cellular stress response and various drug resistance genes, but as reported for FAM129B, UAB36 provides a list of 35 genes whose biology has to be explored to understand the molecular mechanism underlying their correlation with tamoxifen resistance and breast cancer progression.

## Supplementary information

Supplementary files

Supplementary Data 1

Supplementary Data 2

Supplementary Data 3

Supplementary Data 4

Supplementary Data 5

## Data Availability

The drug response data from the GDSC are publicly available in ftp://ftp.sanger.ac.uk/pub/project/cancerrxgene/releases/current_release/GDSC2_fitted_dose_response_25Feb20.xlsx. Expression profile data from GDSC were obtained from https://cancer.sanger.ac.uk/cell_lines/archive-download#:~:text=CosmicCLP_CompleteGeneExpression.tsv.gz. The drug response and expression profile from the CTRP were obtained from https://ctd2data.nci.nih.gov/Public/Broad/CTRPv2.1_2016_pub_NatChemBiol_12_109/ The TCGA-BRCA RNA-Seq data and survival data analyzed in this study were obtained from the GDC. The z-score gene expression relative to all samples, profiled using Illumina HT-12 v3 microarray and survival data for the METABRIC cohort were obtained from the cBioPortal https://www.cbioportal.org/study/summary?id=brca_metabric. The expression data are available in GEO (RRID:SCR_005012) at GSE26459 and GSE9195. The gene sets were obtained from MSigDB. No blinding of data analysis was performed. The predicted IC50 values of tamoxifen for TCGA-BRCA cohort were obtained from https://manticore.niehs.nih.gov/cancerRxTissue/. The TCGA cohort drug treatment data was obtained from https://www.medrxiv.org/content/10.1101/2021.04.30.21251941v2.supplementary-material. The datasets used in this study were downloaded from the public database.

## Code availability

The source code for the presented analysis is available at https://github.com/ds21uab/UAB36-drug-resistance. The statistical analyses can be reproduced with source code and each step mentioned in the workflow R Markdown file along with datasets provided in the GitHub.
