# Supplementary material for: Development of a polygenic score predicting drug resistance and patient outcome in breast cancer
Source: NPJ Precis Oncol. 2024 Oct 2;8:219. doi: 10.1038/s41698-024-00714-7 (PMC11447244; doi:10.1038/s41698-024-00714-7)
Supplement: Supplementary file 1 — Supplementary files [file 41698_2024_714_MOESM1_ESM.pdf]

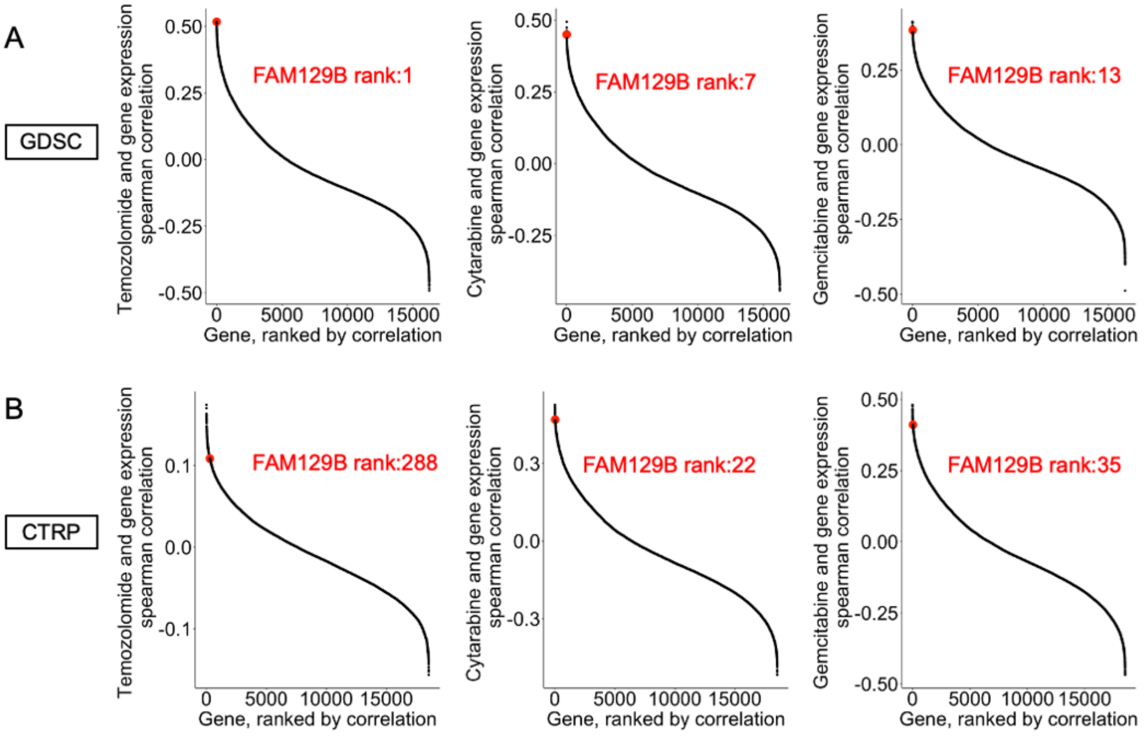

Supplementary Figure 1: FAM129B gene expression ranked as the best candidate gene for drug resistance in cancer cell lines. Correlation of the mRNA Z-score expression for each gene in cancer cell lines with the IC50 to indicated anti-cancer drugs on said cell lines, ranked and plotted from lowest to highest. Temozolomide, Cytarabine, and Gemcitabine across the **A)** GDSC (IC50) and **B)** CTRP (AUC) datasets. Spearman correlation was calculated and ranked for each gene and drug response. Black points: the ranked correlation for each gene. Red point: the ranked correlation for FAM129B gene.

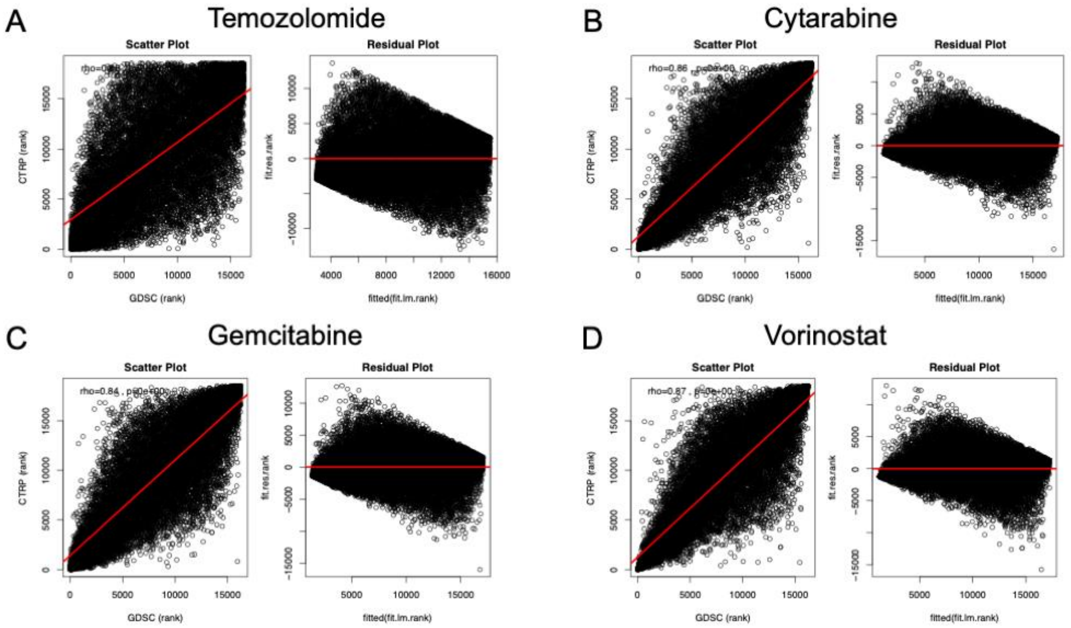

Supplementary Figure 2: Comparison of rank of a gene between GDSC and CTRP. A) Temozolomide; Scatter plot shows correlation of ranks of genes between GDSC and CTRP. Residual plot shows the random scattering of data points around zero (highlighted in red) for the entire range of fitted values. B) Cytarabine. C) Gemcitabine D) Vorinostat. Rho= spearman correlation coefficient; P= pvalue.

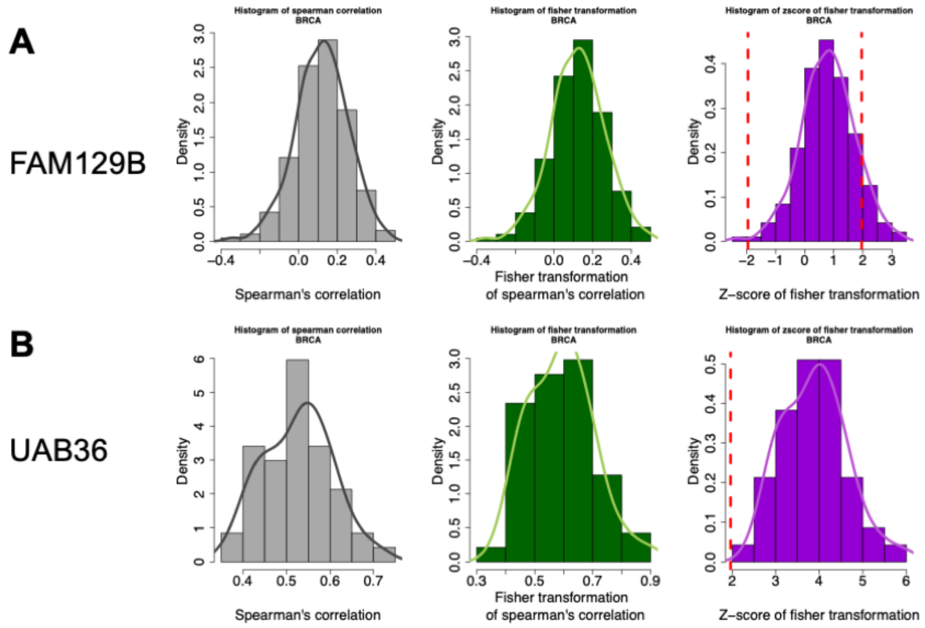

Supplementary Figure 3: Histogram of z-scores after Fisher's Z-transformation in breast cancer cells from the GDSC dataset. A) The bars in grey show the distribution of spearman's rank correlation of FAM129B with FDA approved drugs. The bars in green represents Fisher transformation of the spearman's correlation. The bars in purple represents z-score of the Fisher's transformed correlation. The line in red color shows the cut-off threshold of absolute z-score 1.96. B) Same as A) except for UAB36 in the breast cancer cells.

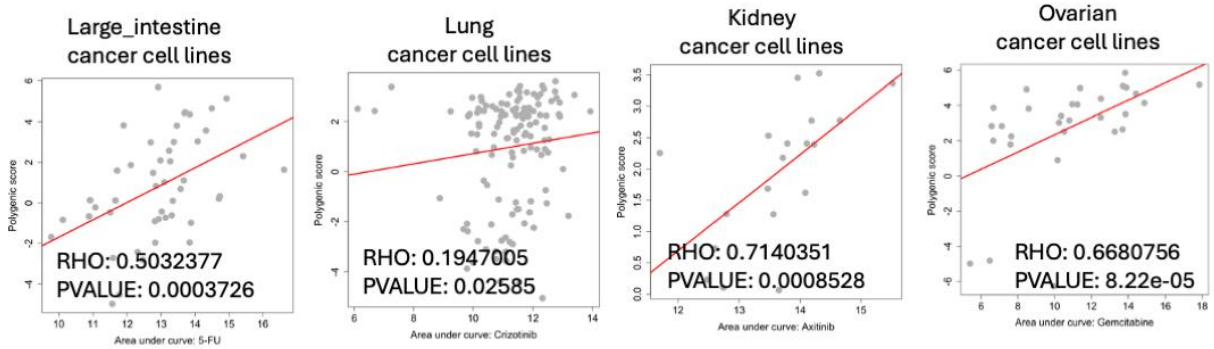

Supplementary Figure 4: Polygenic score based on 36 genes is strongly associated with drug resistance in different cancer types. The response to 5-Fluorouracil in large intestine cancer cell lines, Crizotinib in the lung cancer cell lines, Axitinib in the kidney cancer cell lines, and Gemcitabine in the ovarian cancer cell line are correlated with the UAB36 score.

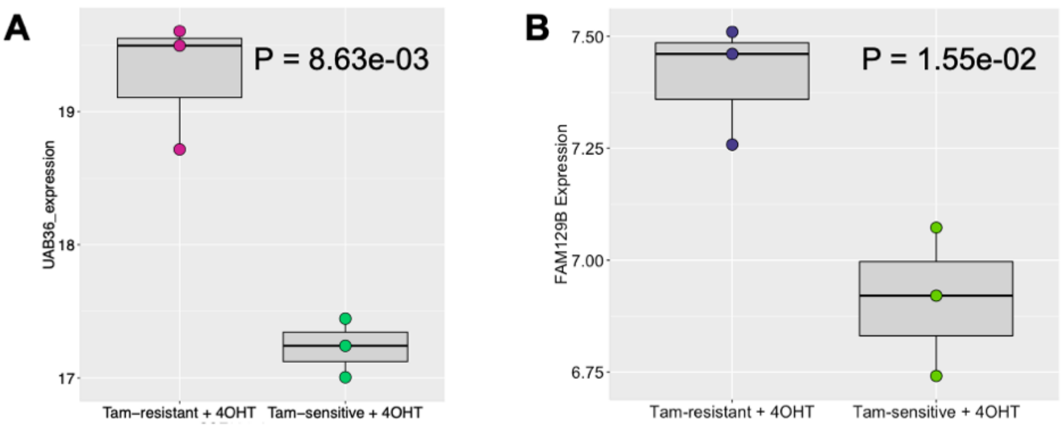

Supplementary Figure 5: UAB36 and FAM129B associated with tamoxifen resistance in GSE26459. (A-B) Boxplot shows UAB36 **(A)** and FAM129B **(B)** expression in MCF7 subclones that were resistant or sensitive to tamoxifen and treated with 4-Hydroxytamoxifen (4OHT). Colors represents MCF7 subclones. The *P* values were obtained from a two-sample t-test.

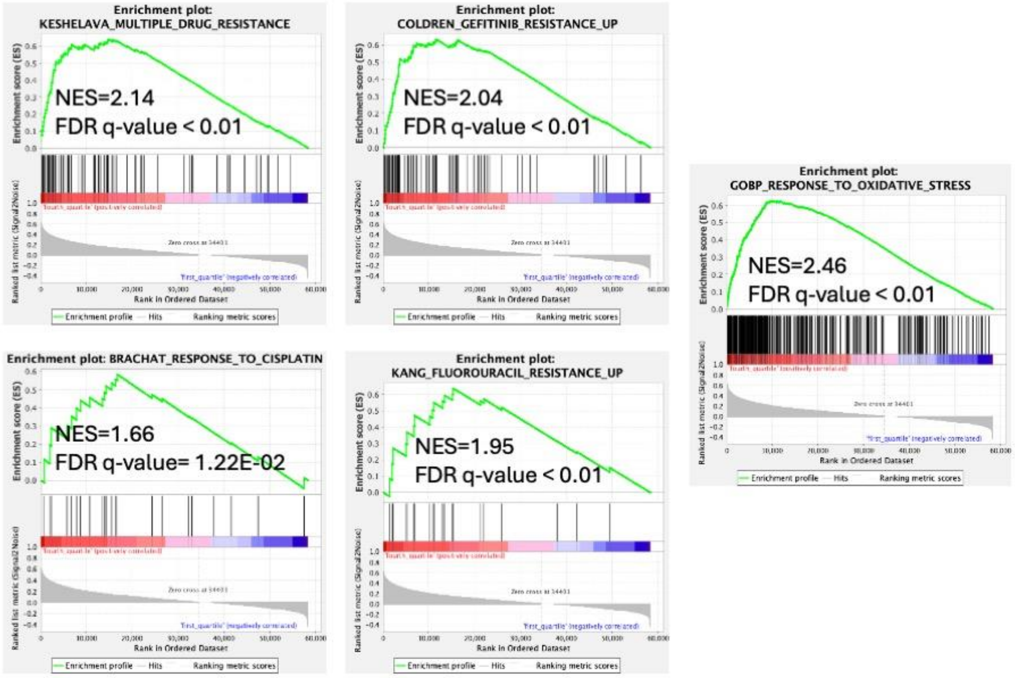

Supplementary Figure 6: GSEA plots shows biological functions enriched in high UAB36 breast cancer tumors. NES= normalized enrichment score, FDR=false discovery rate.

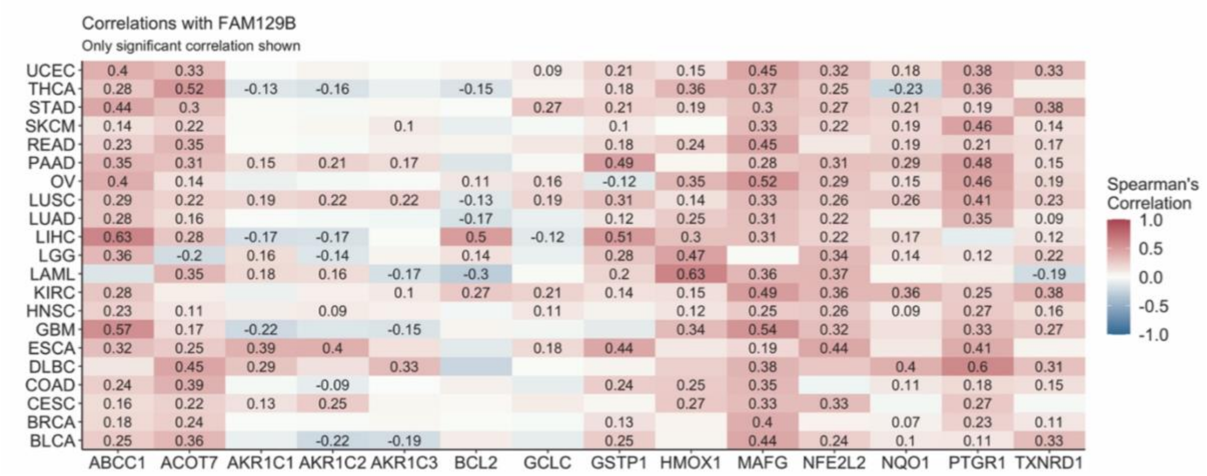

Supplementary Figure 7: FAM129B mRNA expression is positively correlated with Nrf2 targets in different cancer types from the TCGA. The x-axis labels represent NRF2 targets, and the y-axis represents different cancer types. Positive correlations are shown in red color and negative correlation are shown in blue. Only correlation values which are statistically significant are displayed.

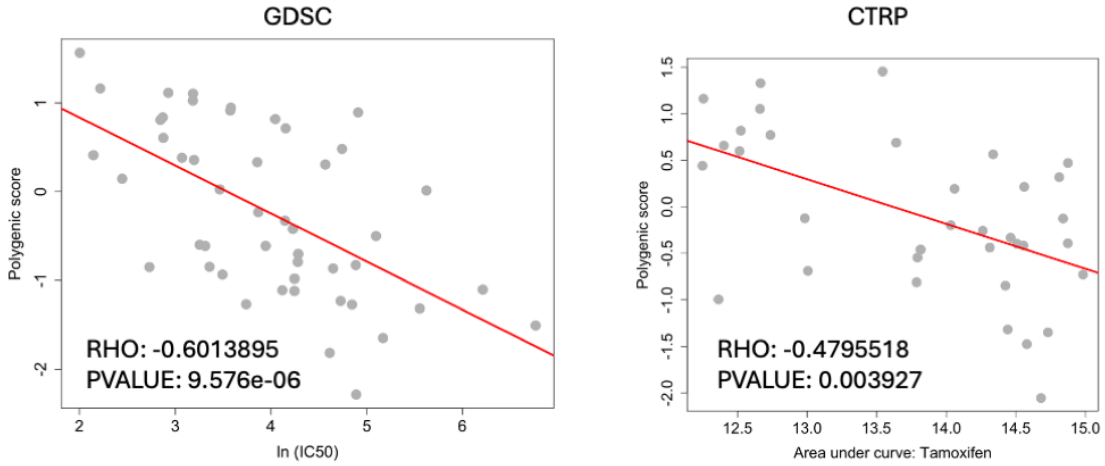

Supplementary Figure 8: Polygenic score based on 5 genes is strongly associated with Tamoxifen sensitivity in breast cancer cells.

Supplementary Table 1. Summary of cancer types included in this study

**Cancer types**	**Tissue sub-type**	**Number of cell lines**	**Number of treated drugs in GDSC**
LUAD	Lung NSCLC adenocarcinoma	53	175
BRCA	Breast	49	190
COREAD	Colorectal Cancer	46	179
SCLC	Small cell lung carcinoma	45	175
SKCM	Melanoma	34	175
ESCA	Esophageal Cancer	32	175
HNSC	Head and Neck carcinoma	27	175
PAAD	Pancreatic Cancer	27	175
GBM	Glioblastoma	26	175
OV	Ovarian Cancer	26	175
ALL	Lymphoblastic Leukemia	24	175
NB	Neuroblastoma	23	175
DLBC	Diffuse large B-cell lymphoma	21	175
LAML	Acute myeloid leukemia	20	175
KIRC	Kidney renal cell carcinoma	19	175
STAD	Stomach adenocarcinoma	19	175
BLCA	Bladder carcinoma	18	175
MM	Myeloma	17	175
LIHC	Liver hepatocellular carcinoma	15	175
LUSC	Lung NSCLC squamous cell carcinoma	15	175
CESC	Cervical Squamous Cell Carcinoma	13	175
THCA	Thyroid Cancer	13	175
LCML	Chronic myeloid leukemia	10	175
LGG	Glioma	9	174
UCEC	Uterine Corpus Endometrial Carcinoma	9	175

Supplementary Table 2: Enrichment of genes in cellular response to stress and cancer related pathways among the list of UAB36 genes.

GSEA pathway	NES	FDR	UAB36 # OF GENES PRESENT	List of UAB36 genes present in the pathway	Reference for drug resistance
GOBP_CELLULAR_RESPONSE_TO_STRESS	2.33	< 0.01	7	CTNNA1, EPHA2, LMNA, MET, NQO1, PLK2, YAP1	PMIDs: 31262713 [1], 35582576 [2]
GOBP_RESPONSE_TO_OXIDATIVE_STRESS	2.46	< 0.01	4	ADAM9, CAPN2, MET, NQO1	PMID: 33805928 [3]
GOBP_CANONICAL_WNT_SIGNALING_PATHWAY	2.31	< 0.01	2	BCL9L, YAP1	PMID: 32258160 [4]
GOBP_EPITHELIAL_TO_MESENCHYMAL_TRANSITION	2.30	< 0.01	1	BCL9L	PMIDs: 28073006 [5], 36271556 [6], 31372302 [7]
GOBP_RAS_PROTEIN_SIGNAL_TRANSDUCTION	2.28	< 0.01	3	ARHGAP29, MET, PLK2	PMID: 33634485 [8]
GOBP_ERBB_SIGNALING_PATHWAY	2.22	1.20E-05	2	BCAR1, GPRC5A	PMID: 28791631 [9]
GOBP_NOTCH_SIGNALING_PATHWAY	2.29	< 0.01	1	YAP1	PMID: 33003540 [10]
GOBP_HIPPO_SIGNALING	2.05	2.67E-04	1	YAP1	PMID: 33467099 [11]

Supplementary References

1 Cheng, K.-C. *et al.* FAM129B, an antioxidative protein, reduces chemosensitivity by competing with Nrf2 for Keap1 binding. *EBioMedicine* **45**, 25-38 (2019). https://doi.org:https://doi.org/10.1016/j.ebiom.2019.06.022

2 Flaherty, R. L., Falcinelli, M. & Flint, M. S. Stress and drug resistance in cancer. *Cancer Drug Resist* **2**, 773-786 (2019). https://doi.org:10.20517/cdr.2019.016

3 Barrera, G. *et al.* Control of Oxidative Stress in Cancer Chemoresistance: Spotlight on Nrf2 Role. *Antioxidants (Basel)* **10** (2021). https://doi.org:10.3390/antiox10040510

4 Yuan, S. *et al.* Role of Wnt/β-Catenin Signaling in the Chemoresistance Modulation of Colorectal Cancer. *Biomed Res Int* **2020**, 9390878 (2020). https://doi.org:10.1155/2020/9390878

5 López-García, C. *et al.* BCL9L Dysfunction Impairs Caspase-2 Expression Permitting Aneuploidy Tolerance in Colorectal Cancer. *Cancer Cell* **31**, 79-93 (2017). https://doi.org:10.1016/j.ccell.2016.11.001

6 Hashemi, M. *et al.* EMT mechanism in breast cancer metastasis and drug resistance: Revisiting molecular interactions and biological functions. *Biomed Pharmacother* **155**, 113774 (2022). https://doi.org:10.1016/j.biopha.2022.113774

7 Song, K. A. & Faber, A. C. Epithelial-to-mesenchymal transition and drug resistance: transitioning away from death. *J Thorac Dis* **11**, E82-e85 (2019). https://doi.org:10.21037/jtd.2019.06.11

8 Healy, F. M., Prior, I. A. & MacEwan, D. J. The importance of Ras in drug resistance in cancer. *Br J Pharmacol* **179**, 2844-2867 (2022). https://doi.org:10.1111/bph.15420

9 Wang, Z. ErbB Receptors and Cancer. *Methods Mol Biol* **1652**, 3-35 (2017). https://doi.org:10.1007/978-1-4939-7219-7_1

10 BeLow, M. & Osipo, C. Notch Signaling in Breast Cancer: A Role in Drug Resistance. *Cells* **9** (2020). https://doi.org:10.3390/cells9102204

11 Zeng, R. & Dong, J. The Hippo Signaling Pathway in Drug Resistance in Cancer. *Cancers (Basel)* **13** (2021). https://doi.org:10.3390/cancers13020318

Supplementary Table 3: Multivariate Cox regression analysis of UAB36, ENDORSE and PAM50 with established clinical risk factors for overall survival in the tamoxifen treated METABRIC ER+/HER2- cohort (n=895)

**Variables**	**HR**	***P***
**UAB36**		
UAB36 *(high vs. low)*	**1.282689**	**0.020601**
AGE *(continuous)*	**1.059365**	**< 2e-16**
TUMOR STAGE	**1.391941**	**0.000652**
TUMOR GRADE	1.142418	0.116453
**ENDORSE**		
ENDORSE *(high vs. low)*	1.074057	0.528271
AGE *(continuous)*	**1.059687**	**< 2e-16**
TUMOR STAGE	**1.403837**	**0.000453**
TUMOR GRADE	1.122439	0.194067
**PAM50**		
PAM50 *(high vs. low)*	1.087208	0.470317
AGE *(continuous)*	**1.059286**	**< 2e-16**
TUMOR STAGE	**1.401654**	**0.000486**
TUMOR GRADE	1.114601	0.233212

[a]In multivariate Cox regression analysis, the gene set median drug score was used as a cutoff to divide patients into high and low score groups.
[b]*P-value* in bold typeface indicates statistical significance ($P < 0.05$).

Description of Additional Supplementary Files

Supplementary Data 1: FAM129B mRNA expression associated with drug resistance through diverse mechanism of actions in the GDSC database

Supplementary Data 2: Correlation of FAM129B with Drug Resistance (IC50) of FDA approved anti-cancer drugs across cancer cell lines in the GDSC database

Supplementary Data 3: Correlation of UAB36 with Drug Resistance (IC50) of FDA approved anti-cancer drugs across cancer cell lines in the GDSC database

Supplementary Data 4: Multivariate Cox Regression analysis of UAB36-1 gene signatures in the METABRIC cohort.

Supplementary Data 5: Multivariate Cox Regression analysis of UAB36-1 gene signatures in the GSE9195 cohort.
